# Five-year outcomes of different techniques for minimally invasive mitral valve repair in Barlow’s disease

**DOI:** 10.1093/ejcts/ezae213

**Published:** 2024-05-23

**Authors:** Leo Pölzl, Can Gollmann-Tepeköylü, Felix Nägele, Kardelen Cetin, Johannes Spilka, Johannes Holfeld, Ulvi C Oezpeker, Luka Stastny, Michael Graber, Jakob Hirsch, Clemens Engler, Julia Dumfarth, Elfriede Ruttmann-Ulmer, Herbert Hangler, Michael Grimm, Ludwig Müller, Daniel Höfer, Nikolaos Bonaros

**Affiliations:** Department of Cardiac Surgery, Medical University of Innsbruck, Innsbruck, Austria; Department of Cardiac Surgery, Medical University of Innsbruck, Innsbruck, Austria; Department of Cardiac Surgery, Medical University of Innsbruck, Innsbruck, Austria; Department of Cardiac Surgery, Medical University of Innsbruck, Innsbruck, Austria; Department of Cardiac Surgery, Medical University of Innsbruck, Innsbruck, Austria; Department of Cardiac Surgery, Medical University of Innsbruck, Innsbruck, Austria; Department of Cardiac Surgery, Medical University of Innsbruck, Innsbruck, Austria; Department of Cardiac Surgery, Medical University of Innsbruck, Innsbruck, Austria; Department of Cardiac Surgery, Medical University of Innsbruck, Innsbruck, Austria; Department of Cardiac Surgery, Medical University of Innsbruck, Innsbruck, Austria; Department of Cardiac Surgery, Medical University of Innsbruck, Innsbruck, Austria; Department of Cardiac Surgery, Medical University of Innsbruck, Innsbruck, Austria; Department of Cardiac Surgery, Medical University of Innsbruck, Innsbruck, Austria; Department of Cardiac Surgery, Medical University of Innsbruck, Innsbruck, Austria; Department of Cardiac Surgery, Medical University of Innsbruck, Innsbruck, Austria; Department of Cardiac Surgery, Medical University of Innsbruck, Innsbruck, Austria; Department of Cardiac Surgery, Medical University of Innsbruck, Innsbruck, Austria; Department of Cardiac Surgery, Medical University of Innsbruck, Innsbruck, Austria

**Keywords:** Barlow’s disease, Mitral valve regurgitation, Minimal invasive surgery, Cardiac surgery

## Abstract

**OBJECTIVES:**

Barlow’s disease is a specific sub-form of mitral valve (MV) disease, characterized by diffuse excessive tissue and multi segment prolapse. The anterolateral mini-thoracotomy represents the standard access for MV regurgitation in many centres. It still remains unclear which surgical technique provides the best results. Therefore, the aim of this study was to compare operative safety and mid-term outcomes after (i) isolated annuloplasty, (ii) use of additional artificial chordae or (iii) leaflet resection in patients suffering from Barlow’s disease undergoing minimally invasive MV repair.

**METHODS:**

A consecutive series of patients suffering from Barlow′s disease undergoing minimally invasive MV surgery between 2001 and 2020 were analysed (*n* = 246). Patients were grouped and analysed according to the used surgical technique. The primary outcome was a modified Mitral Valve Academic Research Consortium combined end-point of mortality, reoperation due to repair failure or reoccurrence of severe mitral regurgitation within 5 years. The secondary outcome included operative success and safety up to 30 days.

**RESULTS:**

No significant difference was found between the 3 surgical techniques with regard to operative safety (*P* = 0.774). The primary outcome did not differ between groups (*P* = 0.244). Operative success was achieved in 93.5% and was lowest in the isolated annuloplasty group (77.1%). Conversion to MV replacement was increased in patients undergoing isolated annuloplasty (*P* < 0.001).

**CONCLUSIONS:**

Isolated annuloplasty, use of additional artificial chordae and leaflet resection represent feasible techniques in Barlow patients undergoing minimally invasive MV surgery with comparable 5-year results. In view of the increased conversion rate in the annuloplasty group, the pathology should not be oversimplified.

## Graphical Abstract

**Figure:**
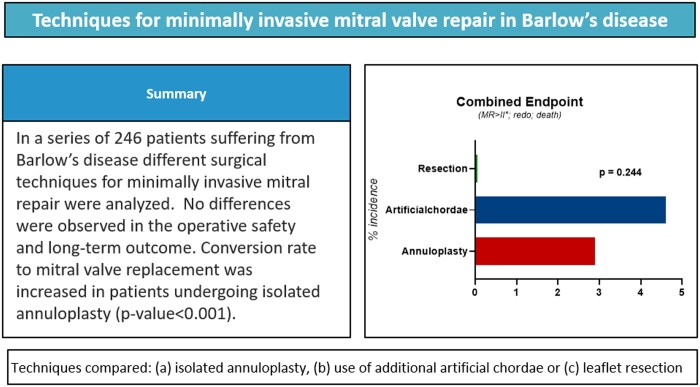

## INTRODUCTION

Mitral valve disease remains the most frequent valvular heart disease [1–3]. Primary mitral regurgitation (MR) due to degenerative leaflet prolapse and/or flail represents the most common aetiology in the Western World [4]. Barlow’s disease is a specific sub-form of degenerative regurgitation, characterized by diffuse excess tissue with or without chordal elongation or functional prolapse, resulting in multi segment prolapse [5–12].

Surgical repair of degenerative mitral valves (MVRe) remains the therapy of choice, exhibiting the best short- and mid-term results [13, 14]. Patients with Barlow’s disease are typically younger and potentially profit even more from MVRe. Due to factors such as variable pathology and tissue quality, bi-leaflet prolapse, risk of systolic anterior motion (SAM) and annular calcifications, MVRe remains a challenging procedure in Barlow patients. Therefore, reported repair rates and postoperative results are surgeon and centre dependent [15].

The anterolateral mini-thoracotomy represents the standard access for MVR in many centres. Due to increased operative times and complexity, standardization of valve repair techniques is crucial to guarantee effective repair rates without increasing the perioperative risk for the patient. Isolated annuloplasty, use of artificial chordae, leaflet resection and edge-to-edge repair are the most common surgical techniques for the repair of Barlow’s disease. New technical advancements including 3D visualization have further improved operative outcomes [16].

However, it remains unclear which surgical technique exhibits the best mid-term results in patients with Barlow’s disease. Therefore, the aim of this study was to compare operative safety and mid-term outcome after (i) isolated annuloplasty, (ii) use of additional artificial chordae or (iii) leaflet resection in patients suffering from Barlow’s disease undergoing minimally invasive mitral repair.

## METHODS

### Ethics statement

This study was performed in accordance with the Declaration of Helsinki, and permission to use anonymized data without patient consent for this study was obtained from the Innsbruck Medical University Institutional Review Board (IRB number 1203/2019).

### Study population and data collection

A consecutive retrospective series of patients undergoing minimally invasive mitral valve surgery (MIMVS) between 2001 and 2020 at the Medical University of Innsbruck were included in this study (*n* = 1128). The indication for surgery was decided upon heart team discussion between cardiologists and cardiac surgeons. All patients underwent MIMVS via anterolateral thoracotomy with use of either 2D or 3D endoscopy. Only patients with Barlow’s disease were included in the final study cohort (*n* = 246). The definition of Barlow’s disease was based on echocardiographic characteristics, confirmed during intraoperative valve analysis, and defined as follows: single or bi-leaflet prolapse with elongated or ruptured chordae, functional prolapse with typical leaflet billowing and presence of excessive tissue or indentations. The surgical technique (annuloplasty alone, additional usage of artificial chordae or leaflet resection) was decided by the surgeon after inspection of the valve. The decision on making how to repair the Barlow valve was mainly based on the phenotype of the disease (Supplementary Material, Table S1). Patients with phenotypes including bi-leaflet billowing and central regurgitation jet underwent mostly ring-only repair. Patients with bi-leaflet billowing and eccentric regurgitation jet or anatomical prolapse due to elongated or ruptured chordae on top of bi-leaflet billowing and excessive valve tissue underwent ring and leaflet repair including either chordal replacement or leaflet resection or both. For annuloplasty, complete semi-rigid rings were used. A standard sizer model was used to determine the correct size of the ring based mainly on the length of the anterior leaflet under consideration of the intertrigonal distance. For chordal replacement, PTFE ((polytetrafluoroethylene) Seramon^®^ Serag-Wiessner, Naila, Germany) loops were used in the majority of the patients. Free-hand artificial chordal replacement was performed by using 4/0 ePTFE sutures (Gore-TEX CV4, Flagstaff AZ). The choice of MVRe technique was independent from TVR.

Patient follow-up (FUP) data were obtained by chart review, patient interviews and echocardiographic FUP. Mortality data were obtained by the Austrian Federal Statistics Institute (‘*Statistik Austria*’). The overall median survival FUP time was 5.97 years with 42% reaching the 5-year FUP time point. The FUP was censored at 5 years resulting with a median FUP time of 5.0 (3.1–5.0) years. One-hundred percent of the patients have at least 1 postoperative echo at FUP.

### Primary outcome and statistical analysis

The primary outcome was a modified derivative of the Mitral Valve Academic Research Consortium (MVARC) criteria including a combination of mortality, reoperation due to repair failure or reoccurrence of severe MR within 5 years (MVARC-event-free survival). The secondary outcome included operative success and safety at 30 days. Operative success was defined as a successful primary mitral repair without conversion to valve replacement or to larger thoracic incisions, no residual MR > mild and no need for reoperation for any reason within the first 30 days. Perioperative safety was defined as freedom from death, perioperative myocardial infarction, stroke, low output syndrome or reoperation for bleeding in the perioperative period (30 days). The outcome data were complete in all patients. The cohort was divided into 3 groups based on the surgical technique used: isolated annuloplasty, additional use of artificial chordae or leaflet resection. Categorical variables are presented as frequencies and proportions. Continuous variables are presented as medians and interquartile range. Differences between groups were assessed by parametric and non-parametric tests as appropriate. Multiple testing was adjusted according to Bonferroni’s method to avoid Type I error inflation. *P*-values were *post hoc* Bonferroni adjusted for multiple testing. FUP data were censored after 5 years. Kaplan–Meier estimates were used to plot survival curves and compared using log-rank test. *P* values <0.05 were considered statistically significant. Attempted and failed repair was a prerequisite for the definition of conversion to mitral replacement. All statistical analyses were performed with SPSS Version 29.0 (IBM Corporation, Armonk, NY, USA), MedCalc Version 20.216 (MedCalc Software Ltd, Ostend, Belgium) and GraphPad Prism version 9.0 (Graph Pad Software Inc., San Diego, CA, USA).

## RESULTS

### Study population

This study included 246 patients with Barlow’s disease who underwent MIMVS. In most patients, a bi-leaflet prolapse was observed intraoperatively (45.2%). In 14.6% of patients, a prolapse of the AML and in 40.2% patients, a prolapse of the PML was found. Isolated annuloplasty was performed in 14%, artificial chordae were used in 62% and mitral leaflet resection was performed in 24% of patients. In 78% of patients, artificial chordae were implanted, and prefabricated loops were used. In median 5 (4–8), artificial chordae were implanted; 25.2% of patients were female median 53 (46–63) years old and both did not differ between the groups. Pre-existing hypertension differed between the groups (57.6 vs 43.9 vs 24.1%; *P*-value = 0.004). Dyslipidaemia was most frequent in patients undergoing isolated annuloplasty (45.5 vs 23 vs 19%; *P*-value < 0.001) and 4.1% patients suffered from diabetes.

Across all groups 4.9% suffered from chronic obstructive pulmonary disease and 8.1% had a history of smoking. Patients had an unrestricted left ventricular function [median left ventricular ejection fraction 60 (55–65)%] and almost all suffered from severe MR (97.7%). The EuroSCORE II was calculated in median at 0.8 (0.6–1.4)% (all Table 1).

**Table 1:** Characteristics of the patients at baseline

Patients characteristics	*n* = 246	Annuloplasty, *n* = 35 (14%)	Artificial chordae, *n* = 153 (62%)	Resection, *n* = 58 (24%)	*P*-value
Age (years)	53 [46–63]	58 [50–67]	53 [46–64]	51 [42–57]	0.020
Sex (female)	62 (25.2%)	11 (31.4%)	36 (23.5%)	15 (25.9%)	0.619
Hypertension	94 (38.2%)	19 (57.6%)	61 (43.9%)	14 (24.1%)	**0.004**
Diabetes	10 (4.1%)	4 (12.1%)	3 (2.2%)	3 (5.2%)	**0.039**
Dyslipidaemia	58 (23.6%)	15 (45.5%)	32 (23.0%)	11 (19.0%)	**0.013**
Smoking	20 (8.1%)	4 (12.1%)	12 (8.6%)	4 (6.9%)	0.696
COPD	12 (4.9%)	2 (6.1%)	6 (4.3%)	4 (6.9%)	0.739
LV-EF (%)	60 [55–65]	60 [55–65]	60 [55–65]	60 [56–65]	0.966
Severe mitral regurgitation	244 (99.2%)	34 (97.1%)	153 (100%)	57 (98.3%)	0.160
EuroScore II	0.8 [0.6–1.4]	0.9 [0.7–1.5]	0.8 [0.6–1.1]	0.7 [0.6–0.9]	0.083

Values are median [interquartile range] or *n* (%). *P*-values (bold < 0.05) show differences between all 3 groups.

COPD: chronic obstructive pulmonary disease; LV-EF: left ventricular ejection fraction.

### Operative characteristics and operative safety

Cross clamp time was 117 (95–143) min in median and did not differed between groups. In 16.1% of patients, a concomitant tricuspid valve repair was performed and differed significantly between groups (31.0 vs 16.9 vs 6.9%; *P*-value = 0.014). Conversion to valve replacement was performed most frequently in patients undergoing isolated annuloplasty (26.6 vs 3.2 vs 0%; *P*-value < 0.001). Across groups, in 1.6% of patients, a conversion to a larger thoracic incision was performed. A mild MR was measured in 29.4% of patients after surgery (*P* = 0.405). Overall operative success was achieved in 93.5%, being lowest in the isolated annuloplasty group (77.1%). One patient (1.7%) undergoing leaflet resection suffered a stroke and 2 patients (1.6%) using artificial chordae suffered from perioperative myocardial infarction; 2.4% had a postoperative low output syndrome. Overall, 4 (1.9%) patients had a revision due to a bleeding and 2 (0.9%) patients died during the hospital stay (1 due to cardiogenic after myocardial infarction and 1 due to respiratory failure). The operative safety characteristics, stroke, myocardial infarction, bleeding, low output syndrome and in-hospital mortality, did not differ individually or combined between the used surgical techniques (all Table 2).

**Table 2:** Characteristics of the surgical procedure and operative safety

	All, *n* = 246	Annuloplasty, *n* = 35 (14%)	Artificial chordae, *n* = 153 (62%)	Resection, *n* = 58 (24%)	*P*-value
Opeartive characteristics					
X-clamp time	117 [95–143]	106 [84–137]	112 [95–136]	131 [99–149]	0.078
Concomitant tricuspid valve surgery	34 (16.1%)	9 (31.0%)	21 (16.9%)	4 (6.9%)	**0.014**
Commissural regurgitation	10 (4.1%)	2 (5.7%)	5 (3.3%)	3 (5.2%)	0.713
Conversion to valve replacement	12 (5.6%)	8 (26.6%)	4 (3.2%)	0 (0%)	**<0.001**
Conversion to larger thoracic incisions	4 (1.6%)	1 (3.3%)	2 (1.6%)	1 (1.7%)	0.820
MR II° upon procedure	0 (0%)	0 (0%)	0 (0%)	0 (0%)	–
MR I° upon procedure	62 (29.4%)	8 (27.6%)	33 (26.6%)	21 (36.2%)	0.405
Redo for early failure	1 (0.5%)	0 (0%)	1 (0.8%)	0 (0%)	0.703
Operative success	230 (93.5%)	27 (77.1%)	146 (95.4%)	57 (98.3%)	**<0.001**
Operative safety					
Stroke	1 (0.5%)	0 (0%)	0 (0%)	1 (1.7%)	0.266
Myocardial infarction	2 (0.9%)	0 (0%)	2 (1.6%)	0 (0%)	0.492
In-hospital mortality	2 (0.9%)	1 (3.4%)	1 (0.8%)	0 (0%)	0.285
Revision due to bleeding	4 (1.9%)	1 (3.4%)	3 (2.4%)	0 (0%)	0.432
Low cardiac output syndrome	6 (2.4%)	1 (3.4%)	4 (3.2%)	1 (1.7%)	0.832
Perioperative safety	233 (94.7%)	33 (94.3%)	144 (94.1%)	56 (96.6%)	0.774

Values are median [interquartile range] or *n* (%); operative success was defined as a successful primary mitral repair without conversion to valve replacement or to larger thoracic incisions, no residual mitral regurgitation (MR) > mild and no need for reoperation for any reason within the first 30 days. Perioperative safety was defined as freedom from death, perioperative myocardial infarction, stroke, low output syndrome or reoperation for bleeding in the perioperative period (30 days). Bold p-values < 0.05.

### Outcome

**Figure 1:**
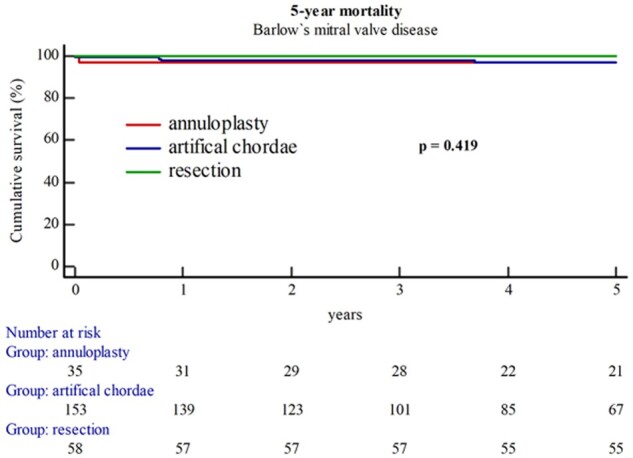
Five-year mortality. Kaplan–Meier estimates show no significant differences in mortality between the 3 surgical techniques.

**Figure 2:**
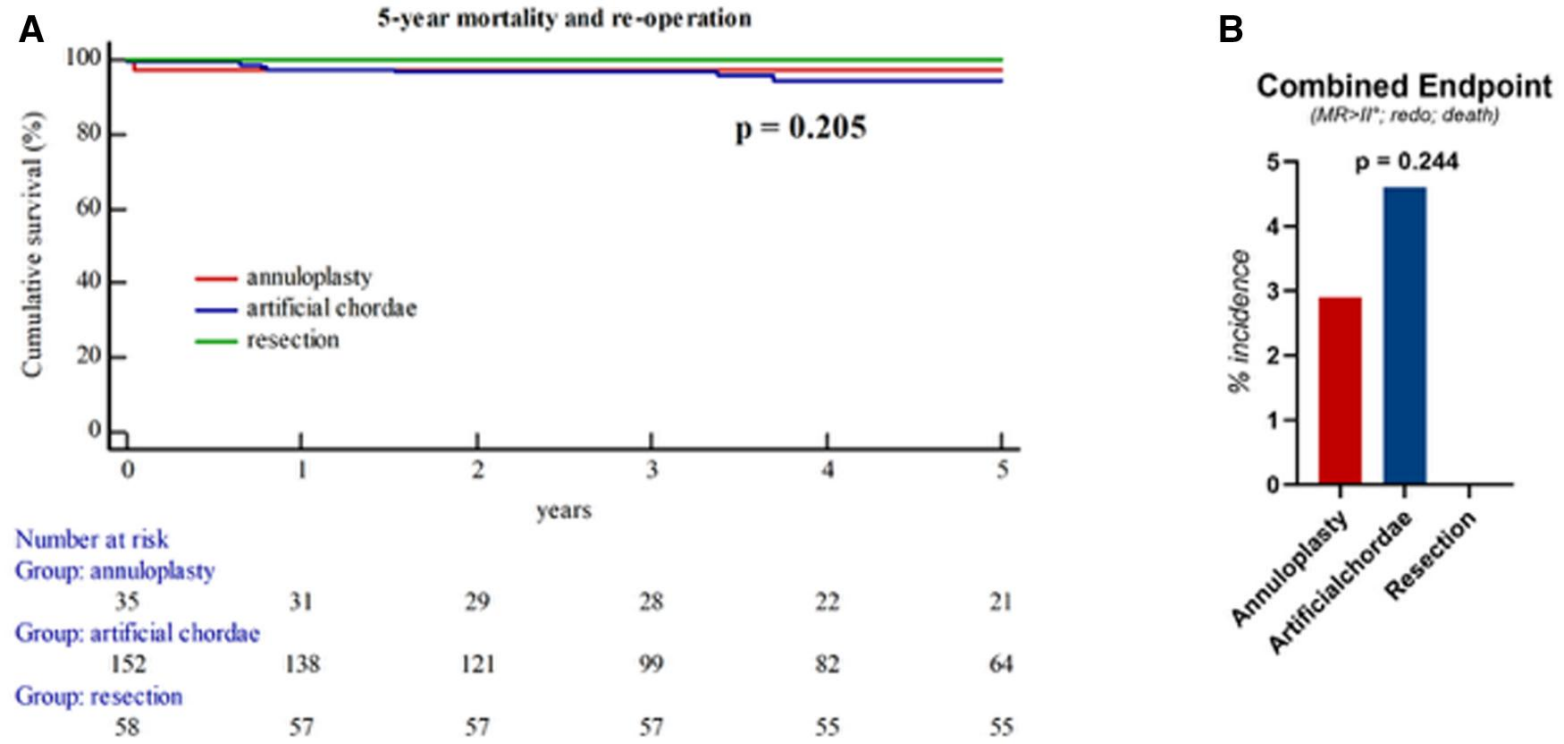
Combined end-points. (**A**) Kaplan–Meier estimates show no significant differences in mortality and freedom of reoperation between the 3 surgical techniques. (**B**) In the combined endpoint, including 5-year mortality, reoperation or reoccurrence of severe mitral regurgitation, no significant differences were observed.

Postoperatively, left ventricular ejection fraction was in median 60 (55–65)% and did not differ between groups. Across groups, patients were intubated for 6 (5–10) h, stayed 20 (18–21) h in the intensive care unit and were discharged from the hospital the 8th (7–9) day after surgery. One patient (1.6%) had postoperatively temporary renal failure (all Table 3). During the mid-term FUP, reoperation due to repair failure was performed in 3 (1.2%) patients. This did not differ significantly across groups (Table 3). One patient (2.9%) in the annuloplasty group and 4 patients (2.6%) in the artificial chordae group died during the 5-year FUP. Kaplan–Meier estimates did not show significant difference in the 5-year mortality between isolated annuloplasty, additional use of artificial chordae and leaflet resection (log-rank: *P*-value = 0.419) (Fig. 1). Even in combination of freedom of reoperation and mortality, no differences were observed during the 5-year FUP (log-rank: *P*-value = 0.205) (Fig. 2A). MVARC-event-free survival, a combined end-point including reoccurrence of severe MR, reoperation due to failure or death within 5 years, did not differ between groups (2.9 vs 4.6 vs 0%; *P*-value = 0.244) (Fig. 2B).

**Table 3:** In-hospital and mid-term outcome

	All, *n* = 246	Annuloplasty, *n* = 35 (14%)	Artificial chordae, *n* = 153 (62%)	Resection, *n* = 58 (24%)	*P*-value
In hospital outcomes					
LV-EF (%)	60 [55–65]	60 [55–65]	60 [55–65]	60 [55–65]	0.936
Intubation time (hours)	6 [5–10]	6 [5–10]	6 [5–9]	7.5 [5–10]	0.402
ICU-stay (hours)	20 [18–21]	20 [17–21]	20 [18–21]	19 [18–21]	0.175
Hospital-stay (day)	8 [7–9]	8 [7–10]	8 [7–9]	8 [7–8]	0.210
Renal failure	1 (1.6%)	0 (0%)	1 (4.0%)	0 (0%)	0.453
Mid-term outcome					
MR > II°	0 (0%)	0 (0%)	0 (0%)	0 (0%)	–
Redo for failure	3 (1.2%)	0 (0%)	3 (2.0%)	0 (0%)	0.397

Values are median [interquartile range] or *n* (%).

ICU: intensive care unit; LV-EF: left ventricular ejection fraction; MR: mitral regurgitation.

## DISCUSSION

This study investigated possible differences in outcomes after minimally invasive mitral valve surgery in patients suffering from Barlow’s disease. Therefore, we aimed to elucidate differences in the operative success and safety and the mid-term outcome after either isolated annuloplasty, use of additional artificial chordae or leaflet resection.

No significant differences were found between the 3 surgical techniques with regard to the primary outcome, MVARC-event-free survival—mortality, reoperation due to repair failure or recurrence of severe MR within 5 years. Operative success, primary mitral repair without conversion to valve replacement or major thoracic incisions, no residual MR > mild and no need for reoperation for any reason within the first 30 days, was achieved in 93.5% and was lowest in the isolated annuloplasty group (77.1%). Within patient characteristics and operative safety, no clinically significant differences were observed. The conversion rate to mitral valve replacement was increased in patients who underwent isolated annuloplasty and was also the cause of the difference in operative success.

Surgical repair remains the treatment of choice for degenerative mitral valve disease [13, 14]. The minimally invasive approach in patients suffering from Barlow’s disease still remains a certain surgical challenge. Data from larger series are scarce [17]. We present the results of a large representative cohort of Barlow patients. Patients were operated by 6 experienced surgeons in a centre within 2 decades of an evolving experience with MIMVS. Therefore, a possible effect of a learning curve does not play a significant role in this analysis [18, 19].

Which is the right technique to be used is still a matter of debate. Differences between the 3 commonly used techniques, isolated annuloplasty, use of additional artificial chordae or leaflet resection, have not been investigated yet. We were able to demonstrate the feasibility of complex techniques using a minimally invasive and endoscopic access with very good mid-term results comparable with other series [17, 20–22]. Within this study, we could observe an increased rate of conversion to valve replacement in patient’s with isolated annuloplasty. Of the 8 patients who were converted to valve replacement, 1 patient had severe SAM after repair. In the other 7, the repair failed for individual valve characteristics such as calcifications, extensive fibrosis or other phenotypes of complex anatomies. The simple ring annuloplasty is propagated for MIMVS [23, 24]. However, in view of the increased conversion rate with this technique and the risk of SAM, we believe that this should not be oversimplified. Due to the long study period, the echocardiographical evaluation in terms of risk of SAM has not been unified. Modern evaluation techniques include the mobility of the anterior leaflet, the C-sept distance and the aorto-mitral angle. These parameters provide significant information on the risk of SAM post-repair and are included to the selection of the repair technique [25, 26].

In only 16% of patients, the tricuspid valve also required intervention. We hypothesize that this is due to the fact that Barlow patients come to surgery earlier, and therefore tricuspid regurgitation secondary to MR is rarer than in other pathologies. No other clinically significant differences were observed in the surgical characteristics or mid-term outcome.

We think that on the basis of these results, it would be wrong to believe that all 3 techniques work equally well in all the patients. MVRe surgery requires a great amount of surgical experience, especially in assessing the valves and the underlying pathomechanism. This counts even more in patients suffering from Barlow’s disease. The challenge, or rather the difficulty, is probably to use the right technique for the right patient or diagnosis. This study demonstrates that the right technique used in the right patient results in a good mid-term outcome. It is very difficult to objectify which patient-related factors require which technique. We have to accept that we do not know up to a certain point which factors require certain repair techniques. Within this study, edge-to-edge repair, as the ‘*Alfieri stitch*’, was not analysed since it was not performed in the analysed patients. However, this is a very successful technique for pathological conditions that are prone to systolic anterior movement of the anterior leaflet. Further studies have to be performed to take edge-to-edge techniques into account.

The repair of this very complex mitral valve pathology still requires a high level of surgical experience. In order to objectify this experience, further studies on a larger number of cases are necessary.

### Limitations

This is a single centre retrospective observational study and has the corresponding limitations. However, all patients who underwent minimally invasive mitral valve surgery were consecutively included in a specific time period. In none of the patients, an edge-to-edge was performed. Therefore, this represents a limitation of the study. The annuloplasty only group is rather small, and therefore it cannot be ruled out that the group was underpowered in certain statistical analyses. Due to the retrospective nature of this study, some preoperative patient data are not available and missing. This poses a limitation for all evaluations regarding baseline characteristics. The data for the outcomes and end-points of the study were available for all patients. However, the cohort and especially the event rate were small and the results were not adjusted for any possible confounders, which represents a limitation. Therefore, the results should be interpreted with caution.

## CONCLUSION

Isolated annuloplasty, use of additional artificial chordae and leaflet resection represent feasible techniques in Barlow patients undergoing MIMVS with comparable 5-year results when used in the right patients. In view of the increased conversion rate in the annuloplasty group, the pathology should not be oversimplified. Further studies have to be performed to take other techniques, as edge-to-edge repair, into account.

## Supplementary Material

ezae213_Supplementary_Data

## Data Availability

The data underlying this article will be shared on reasonable request to the corresponding author.

## Notes

Presented at EACTS Annual Meeting 2023, Vienna, Austria.

## ABBREVIATIONS

FUP: Follow-up
MIMVS: Minimally invasive mitral valve surgery
MR: Mitral regurgitation
MVRe: mitral valve repair
MVARC: Mitral Valve Academic Research Consortium
SAM: Systolic anterior motion
