# Corrigendum to: Five-year outcomes of different techniques for minimally invasive mitral valve repair in Barlow’s disease

**DOI:** 10.1093/ejcts/ezae279

**Published:** 2024-07-22

This is a corrigendum to: Leo Pölzl, Can Gollmann-Tepeköylü, Felix Nägele, Kardelen Cetin, Johannes Spilka, Johannes Holfeld, Ulvi C Oezpeker, Luka Stastny, Michael Graber, Jakob Hirsch, Clemens Engler, Julia Dumfarth, Elfriede Ruttmann-Ulmer, Herbert Hangler, Michael Grimm, Ludwig Müller, Daniel Höfer, Nikolaos Bonaros, Five-year outcomes of different techniques for minimally invasive mitral valve repair in Barlow’s disease, *European Journal of Cardio-Thoracic Surgery*, Volume 65, Issue 6, June 2024, ezae213, https://doi.org/10.1093/ejcts/ezae213.

In the originally published version of the manuscript, there was an error in section **Study population and data collection**; in the penultimate sentence, text in the parentheses should read: “[…]Serag-Wiessner[…]” instead of: “[…]Serrag-Wiesner[…]”.

The emendation has been made to the article.

