# Supplementary material for: Five-year outcomes of different techniques for minimally invasive mitral valve repair in Barlow’s disease
Source: Eur J Cardiothorac Surg. 2024 May 23;65(6):ezae213. doi: 10.1093/ejcts/ezae213 (PMC11150856; doi:10.1093/ejcts/ezae213)
Supplement: ezae213_Supplementary_Data [file ezae213_supplementary_data.docx]

**Supplemental Material to *“****Five-year outcomes of different techniques for minimally invasive mitral valve repair in Barlow’s disease”*

**Supplemental Table 1:** Factors influencing decision making on surgical treatment of Barlow’s disease

|  | **Isolated annuloplasty** | **Annuloplasty + artificial chordae** | **Annuloplasty +/-artificial chordae + resection** |
| --- | --- | --- | --- |
| *Central regurgitation* | ***+*** | ***-*** | ***-*** |
| *Eccentric regurgitation* | ***-*** | ***+*** | ***+*** |
| *Anatomical prolapse* | ***-*** | ***+*** | ***+*** |
| *Functional prolapse* | ***+*** | ***+/-*** | ***-*** |
| *Leaflet flail* | ***-*** | ***+*** | ***+*** |
| *Increased risk of SAM* | ***-*** | ***+*** | ***++*** |
| *Annular/Leaflet calcification* | ***-*** | ***-*** | ***++*** |
